# Charcot-Bouchard Aneurysm Diagnosed with CTA and MRA

**DOI:** 10.5334/jbsr.1934

**Published:** 2021-03-12

**Authors:** Sanaa Jamali, José Géraldo Ribeiro Vaz, Guido Wilms

**Affiliations:** 1 Cliniques Universitaires Saint Luc, BE

**Keywords:** Charcot-Bouchard aneurysm, basal ganglia hematoma

## Abstract

Hemorrhage in the basal ganglia resulting to lenticulostriate artery aneurysm rupture is extremely rare. This distal micro-aneurysm of the perforating lenticulostriate arteries is called Charcot-Bouchard aneurysm. We wish to report a case of an hematoma in the basal ganglia due to a Charcot-Bouchard aneurysm demonstrated by Computed Tomography Angiograpy (CTA) and Magnetic Resonance Angiography (MRA) and confirmed by selective catheter angiography.

**Teaching Point:**

Charcot-Bouchard aneurysm is a very rare distal micro-aneurysm of the perforating lenticulostriate arteries. Young patients who experience basal ganglia hemorrhage should have contrast-enhanced CT, especially if they don’t have arterial hypertension and if subarachnoid hemorrhage is associated.

## Case Report

**Figure 1:**
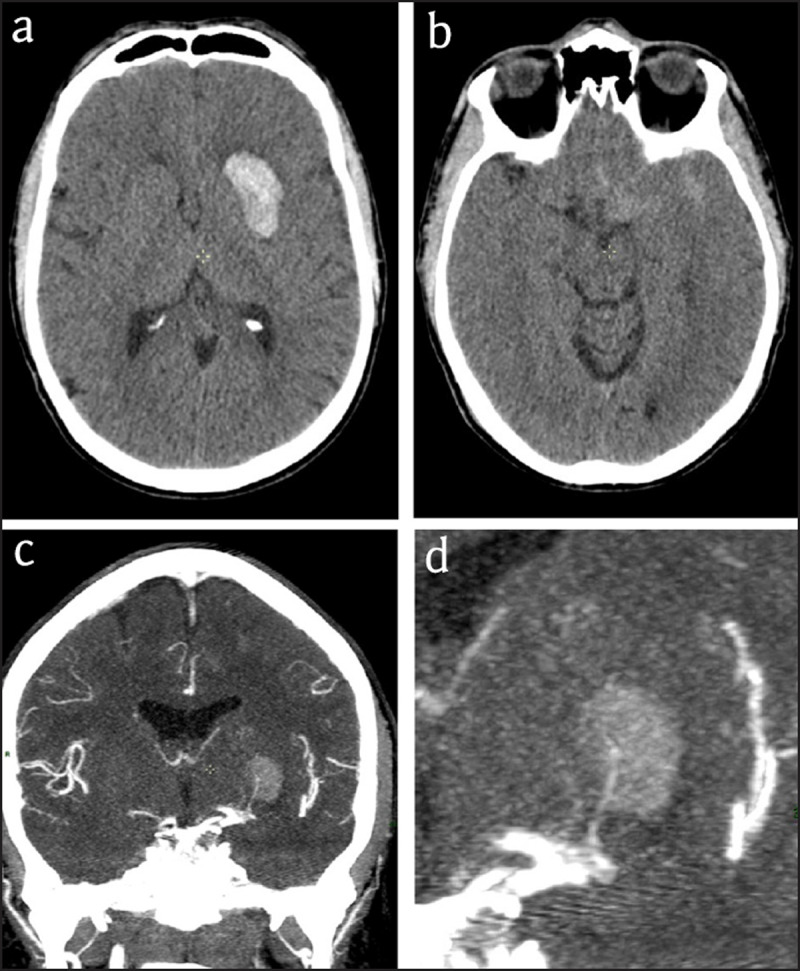
**CT. (a)** Plain CT: Hemorrhage in the left basal ganglia (Arrow) **(b)** Plain CT at a lower level: Notice subarachnoid hemorrhage in the cistern of the middle cerebral artery and the insula (arrowheads) **(c)** CTA: Notice fusiform aneurysm (arrow) on a distal lenticulostriate artery. **(d)** Magnified view of CTA showing the aneurysm in continuity ith the lenticulostriate artery (arrow).

**Figure 2:**
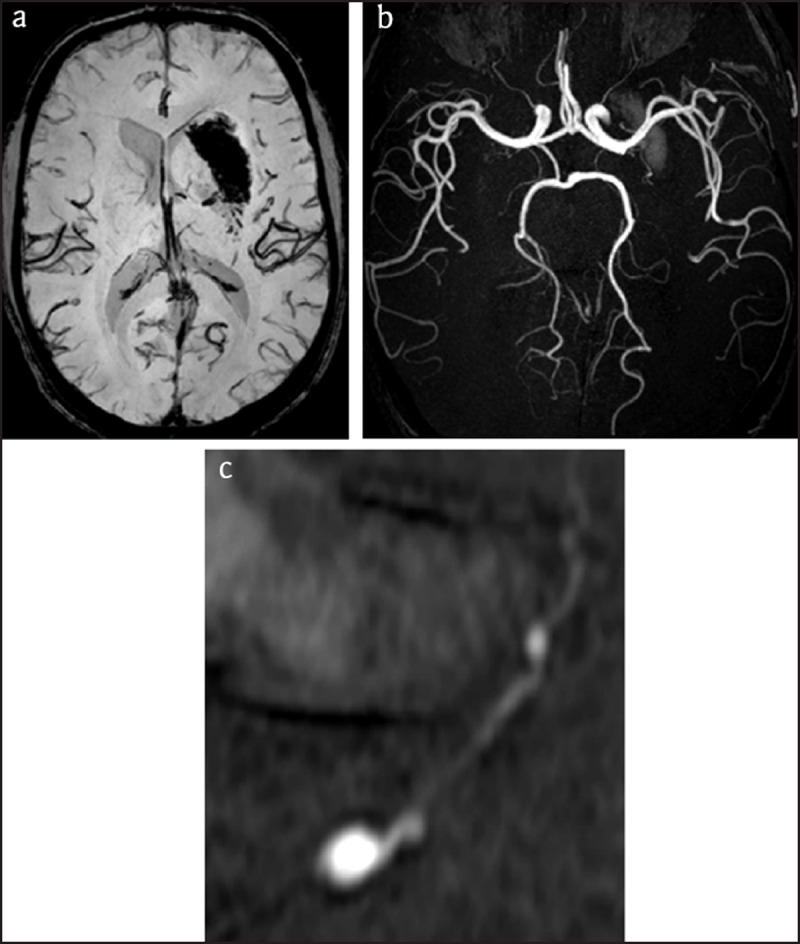
**MRI. (a)** SWI sequence of MRI: Susceptibility artifacts in the left lentiform nucleus due to hemorrhage. **(b)** MRA with TOF sequence: Notice aneurysm as a dense spot (arrow) in the T1 hyperintense hemorrhage. **(c)** MIP of MRA with reconstruction: Notice fusiform dilatation of distal lenticulostriate artery (arrow).

**Figure 3:**
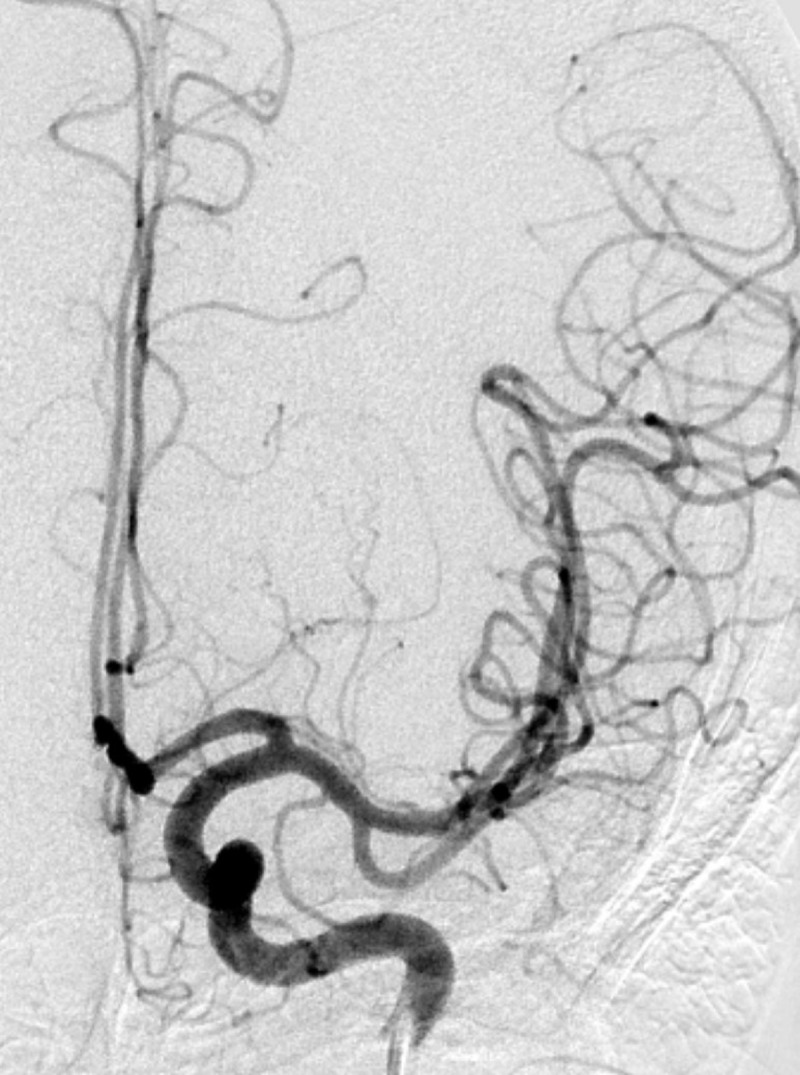
Selective catheter angiography: Fusiform Charcot-Bouchard aneurysm on distal lenticulostriate artery (arrow).

A 47-year-old male patient with a known history of arterial hypertension and migraine suffered a sudden right facio-brachial paresis and dysarthria. National Institutes of Health Stroke Scale (NIHSS) score was 6. Emergency computed tomography (CT) showed a recent hemorrhage in the left basal ganglia (***Figure 1a***) with some subarachnoid hemorrhage in the left insula and the cistern of the middle cerebral artery (***Figure 1b***). CT angiography (CTA), performed because of the young age of the patient and the presence of subarachnoid blood, showed a small distal lenticulostriate aneurysm (***Figures 1c*** and ***1d***). This was confirmed a few days later by magnetic resonance angiography (MRA) (***Figure 2***) and finally by selective left carotid angiography (***Figure 3***). Because of the distal location of the aneurysm it was decided to remain conservative. During the following weeks there was good recuperation of the clinical symptoms with a persistent slight facial asymmetry. Patient is followed up yearly by MRA.

## Discussion

Basal ganglia hemorrhages in patients with chronic arterial hypertension are frequent. In this context, intravenous contrast is not routinely administered for CT. In our patient, the simultaneous presence of subarachnoid blood in the insular cistern and his young age triggered CTA, which allowed us to detect the small Charcot-Bouchard aneurysm. To date, only approximately 60 cases of Charcot-Bouchard aneurysms are reported. They mostly present at a younger age with a mean of 41.9 years [1], and not all patients are hypertensive. The primary type of distal lenticulostriate aneurysms is probably dissecting or pseudo-aneurysms, but true saccular aneurysms exist [1]. They mostly lead to solitary hematomas in the basal ganglia mimicking hypertensive hemorrhage [2]. The hematoma in the basal ganglia may be accompanied by subarachnoid hemorrhage as in our case.

Diagnosis of Charcot-Bouchard aneurysms now can be based on CTA showing an enhancing spot in the hematoma. This so-called spot sign is important to recognize, as it indicates active bleeding and predicts rapid hematoma expansion. The lesion can also be shown on MRA [1] or contrast-enhanced T1-weighted MR, In some cases, however, cross-sectional imaging may fail to display the lesion, which makes catheter angiography the diagnostic standard of reference [1].

Treatment of Charcot aneurysms would be indicated if the aneurysm is saccular or exceeds 5 mm [1]. Treatment options include surgery or embolisation. Conservative management can be considered if the aneurysm is small and fusiform as in our case. The aneurysm will progressively disappear on follow-up imaging in the following years.

To conclude, as there is no difference on non-contrast CT between Charcot aneurysm and hemorrhage without aneurysm, we recommend to carefully perform CTA when the patient is young, when subarachnoid blood is present and/or when there is no context of arterial hypertension.
